# Collaboration and Decision-Making on Trauma Teams: A Survey Assessment

**DOI:** 10.5811/westjem.2020.10.48698

**Published:** 2021-01-11

**Authors:** Kinjal N. Sethuraman, Wan-Tsu W. Chang, Amy L. Zhou, Boyan Xia, Daniel B. Gingold, Maureen McCunn

**Affiliations:** * University of Maryland School of Medicine, Department of Emergency Medicine, Baltimore, Maryland; † University of Maryland School of Medicine, Program in Trauma, Baltimore, Maryland; ‡ University of Maryland, College Park, Maryland; § University of Maryland School of Medicine, Department of Anesthesiology, Baltimore, Maryland

## Abstract

**Introduction:**

Leadership, communication, and collaboration are important in well-managed trauma resuscitations. We surveyed resuscitation team members (attendings, fellows, residents, and nurses) in a large urban trauma center regarding their impressions of collaboration among team members and their satisfaction with patient care decisions.

**Methods:**

The Collaboration and Satisfaction About Care Decisions in Trauma (CSACD.T) survey was administered to members of ad hoc trauma teams immediately after resuscitations. Survey respondents self-reported their demographic characteristics; the CSACD.T scores were then compared by gender, occupation, self-identified leader role, and level of training.

**Results:**

The study population consisted of 281 respondents from 52 teams; 111 (39.5%) were female, 207 (73.7%) were self-reported White, 78 (27.8%) were nurses, and 140 (49.8%) were physicians. Of the 140 physician respondents, 38 (27.1%) were female, representing 13.5% of the total surveyed population. Nine of the 52 teams had a female leader. Men, physicians (vs nurses), fellows (vs attendings), and self-identified leaders trended toward higher satisfaction across all questions of the CSACD.T. In addition to the comparison groups mentioned, women and general team members (vs non-leaders) gave lower scores.

**Conclusion:**

Female residents, nurses, general team members, and attendings gave lower CSACD.T scores in this study. Identification of nuances and underlying causes of lower scores from female members of trauma teams is an important next step. Gender-specific training may be necessary to change negative team dynamics in ad hoc trauma teams.

## INTRODUCTION

Collaboration and communication are recognized factors in successful team dynamics. On trauma teams, leadership, task completion, and delegation are additional characteristics vital to success.^1^ Gender differences in team leadership in acute care settings have not been well studied. Speck and associates reported that male leaders at an academic trauma center perceived themselves as teachers and educators more often than female leaders.^2^ Additionally, based on data from 42 intensive care units, Shortell and colleagues demonstrated that improved physician–nurse communication was associated with better patient outcomes and higher patient and family satisfaction.^3^ In a survey of emergency department (ED) clinicians, Rosenstein and Naylor found unclear roles and responsibilities to be a contributing factor in ineffective communication.^4^ None of these studies, however, addressed communication or collaboration by gender. Emergency departments and trauma resuscitation units must maximize effective collaboration to prevent dangerous and life-threatening situations for their patients.^5^,^6^

Trauma teams are ad hoc assemblages of attending physicians, fellows, residents, nurses, technicians, and medical students who come together for the initial assessment and immediate treatment of a trauma patient.^7^ These teams are generally very effective at treating patients, but team members may have different perceptions of collaboration and communication.^8^,^9^ To our knowledge, this is the first study of collaboration within ad hoc trauma teams from the viewpoint of their members. We also specifically studied gender differences in responses.

The purpose of this study was to investigate trauma team members’ perceptions of collaboration, communication, and leadership and their satisfaction with patient care decisions. We hypothesized that team members have differing views on collaboration during resuscitation, leading to inconsistent levels of satisfaction, and that role, level of responsibility, and gender contribute to these differing views.

## METHODS

### Setting

We conducted this study at a regional Level I trauma center designated for the resuscitation and stabilization of critically ill and injured patients. The center has more than 7000 trauma patient encounters per year. This study was approved by the institutional review board at the university where the trauma center is located.

### Participants

Trauma team members involved in resuscitations were enrolled prospectively, as a random sample, between 2014–2016.

### Survey Methodology

Research assistants spent at least 40 hours per week surveying team members in the trauma center. They conducted the surveys at various times of day and on weekends over two 10-week periods in summer 2015 and summer 2016. New research assistants were trained on the methods of survey administration and collection by the lead investigator. Following completion of a trauma resuscitation, research assistants surveyed at least half of the members of that trauma team; participation was voluntary. On rare occasions, team members declined to participate in the study.

Gender identity, ethnicity, age, occupation, and team role (leader vs non-leader or general team member) were self-reported by participants. Gender options were binary: male and female. Race or ethnicity was self-reported and respondents were given the following ethnicity/race options to choose from: African American, Asian American, Hispanic, Native American, White, or other. Team leaders were typically a senior resident or fellow at the study site.

**Population Health Research Capsule**

What do we already know about this issue?

*Collaboration and communication are critical for successful teams and patient outcomes. Ad hoc trauma teams are effective in patient care but may have different perceptions of team dynamics*.

What was the research question?

How do trauma team members’ perceptions of collaboration, communication, and satisfaction with patient care decisions differ?

What was the major finding of the study?

*Gender, occupation, and team leadership affect perceptions of collaboration and satisfaction among trauma team members*.

How does this improve population health?

*Recognizing differences in perceptions of ad hoc team dynamics allows targeted improvements in collaboration and communication, which ultimately improves the care of trauma patients*.

**Figure 1:**
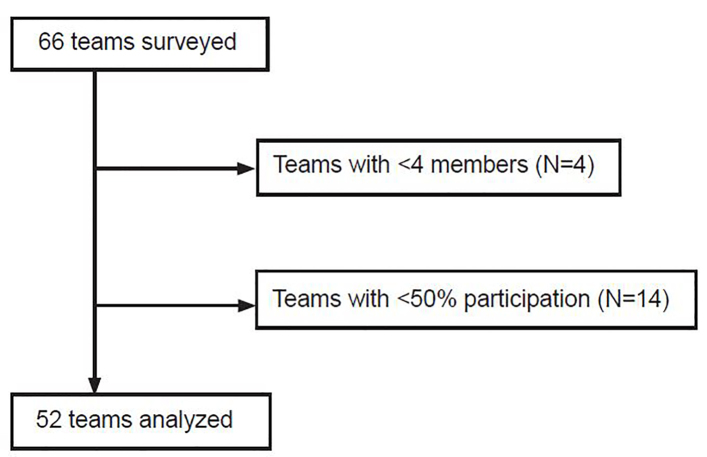
Flow diagram of trauma teams’ eligibility to be included in study analysis.

We excluded teams with fewer than four members or less than 50% of team members participating in the survey (Figure 1) because, at the study site, trauma teams typically consist of nine or more people. Demographic information from surveyed team members was used to calculate the gender, ethnicity, and occupational composition of the team. The team score for each survey question was the mean of the individual responses to the question. Overall team score was the mean of the individual overall scores.

### Collaboration and Satisfaction About Care Decisions in Trauma (CSACD.T) Instrument

The original Collaboration and Satisfaction About Care Decisions instrument has been validated for assessment of nurse–physician collaboration and satisfaction with patient care decisions.^10^ It consists of nine questions measured on a seven-point Likert scale that ask about cooperation, assertiveness, shared responsibility for planning, shared decision-making, open communication, and coordination as important attributes of collaboration.^11^ We chose this instrument for this study because of its excellent internal consistency (Cronbach α = 0.95) and because it has been tested on both physicians and nurses.^11^

The questions were reframed slightly to reflect the trauma setting, creating the CSACD.T (Appendix). The specific questions changed were 1, 8, and 9. A qualifier of “in the trauma bay” was added at the end of Questions 1 and 8 to reflect the location of the resuscitation. Question 8 was changed from “How satisfied were you with the way this decision was made for this patient?” to “How satisfied are you with the overall collaboration between physicians and nurses in the trauma bay?” Question 9 was reworded from “How satisfied were you with the decisions made for the patient?” to “How satisfied are you with collaboration on the trauma service overall?” The total participant satisfaction score is the sum of the scores from the nine questions.

### Statistical Analysis

We compared relative frequencies of categorical variables using chi-squared and Fisher’s exact test. Mean scores were compared between two categorical groups using the Mann-Whitney *U* test. Mean scores were compared across multiple groups using the Kruskall-Wallis test. We calculated individual total scores by summing an individual’s responses from Questions 1–9 of the CSACD.T. The Pearson correlation coefficient was used to determine the linear correlation between a team’s score and percentage of female members. To test for differential effects of gender on satisfaction scores among different occupations and leadership roles, we performed a linear regression with total satisfaction score as the dependent variable and independent variables of gender, occupation, and leadership roles, as well as two- and three-way interaction terms. Survey data were recorded in Microsoft Excel 2016 (Microsoft Corporation, Redmond, WA), and statistical analysis was performed using SAS University Edition Studio version 3.5 (SAS Institute Inc., Cary, NC.).

## RESULTS

Our study group consisted of 281 survey participants from 52 teams. Table 1 presents their demographic information: 39.5% were female and most were between the ages of 20–40 (consistent with the typical age group for residents, fellows, and junior attendings). Most respondents (73.7%) self-identified as White. Half (49.8%) of survey respondents were physicians (attendings, fellows, and residents), and 27.8% were nurses. Team leaders were less likely to be female than non-leaders (24.6% vs 44.3%, *p* = 0.004), and physicians were less likely to be female (27.1%) compared to registered nurses (61.5%) and those with other roles (39.7%, *p* < 0.001).

Table 2 lists the characteristics of the ad hoc trauma teams. Nine (17.3%) of the 52 teams had a female leader. Although we made an effort to survey the team leaders, 14 (26.9%) teams did not have a self-identified leader participate in the survey. The leader might have gone directly from the trauma resuscitation unit to the operating suite or declined to participate in our study. Seventeen teams (32.7%) had more than one self-identified leader; on 15 teams (28.8%), a resident as well as another resident, a fellow, an attending, or a nurse identified themselves as the leader. In these situations, a fellow or an attending could have been supervising a resuscitation that a resident was leading. Teams with more than one self-identified leader gave higher overall mean [SD] team scores compared with teams with no leader or one leader (54.6 [1.6] vs 51.8 [1.9], *p* = 0.03).

**Figure 2:**
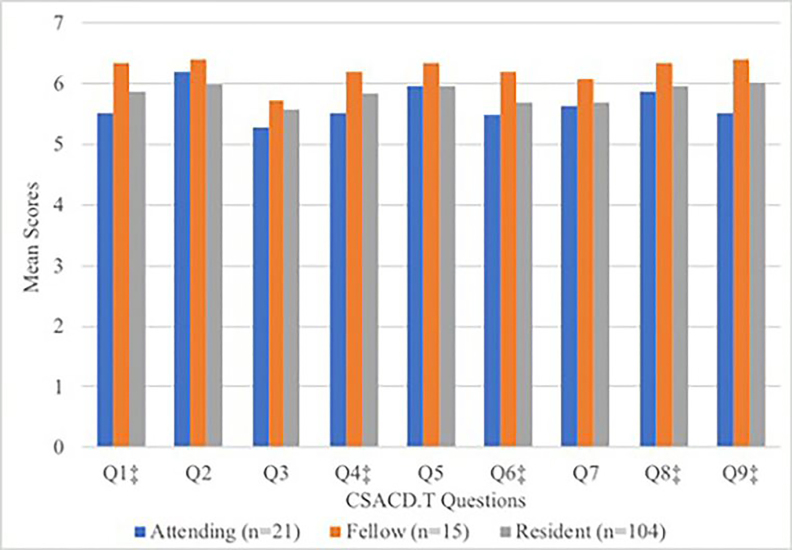
Differences in Collaboration and Satisfaction About Care Decisions in Trauma survey: mean scores* by physician level of training. * Scores based on a 7-point Likert scale. ‡ Denote P value < 0.05.

Male respondents, physicians, and self-identified leaders gave higher scores on almost every question compared with females, non-physicians, and general team members, respectively (Table 3). A higher proportion of team members being female was weakly correlated with lower overall team satisfaction scores (*r*^2^ = 0.14). Difference in overall team score was not statistically significant between male and female leaders (53.9 vs 51.6 [*p* = 0.2]). Thirty male nurses and 48 female nurses completed the survey. The raw scores on most questions of the CSACD.T instrument were higher for male nurses than female nurses. When comparing CSACD.T scores among physicians based on level of training (Figure 2), the scores suggested that, generally, fellows were most satisfied and attendings were least satisfied with team collaboration.

The results of the linear regression containing interaction terms for gender, occupation, and leader showed that there was no significant difference in average total scores between female and male physician leaders (difference 1.47, *p* = 0.52) or between female and male nurse team members (difference 1.23, *p* = 0.52).

## DISCUSSION

This inquiry revealed interesting patterns of perceptions of satisfaction and collaboration in a trauma setting. Physicians gave higher overall scores than did nurses. Steinemann and colleagues surveyed trauma nurses and surgeons and found that those groups had different perceptions of their responsibilities in trauma resuscitations.^12^ Our results indicate a trend toward a greater overall level of satisfaction with care decisions and collaboration between physicians compared with nurses and nursing/medical students, as well as between males compared with females. Prior research has also revealed significant disparities between nurses’ and physicians’ perceptions of teamwork and communication, possibly based in the traditional differences in power and authority between the two occupations.^13^,^14^

Fellows in our study were more satisfied with overall team collaboration than were attendings. There could be several reasons for this finding. Fellows are often making decisions and performing the most critical procedures, while attendings tend to supervise the resuscitation and intervene only when necessary. Attendings are also responsible for teaching and so may have a more critical eye on how the resuscitation is carried out. We also found that teams with more than one self-identified leader gave higher CSACD.T scores than those with a single leader, which could be related to improved communication and collaboration among team members and between physicians and nurses.^15^ Based on these data, we speculate that if multiple team members are assigned to be co-leaders, the perception of collaboration by all of the team members may increase. Alternatively, since leaders had higher scores, the entire team score may be artificially increased. At the study site, trauma team leaders can change with each resuscitation.

The composition of the team can also change as new members rotate on and off a team. Such staffing changes—a feature of ad hoc teams—might play a role in team members’ scores, depending on when the surveys were administered. For example, perceptions within a team may be different at the start of a rotation with team members newly working together compared with a team that has worked together for a number of resuscitations. In future studies, adding trained independent observers to monitor the trauma team will add objective measures of collaboration. Adding time variables—time of day, day of rotation, and number of resuscitations on a given shift—may lead to further insights on team dynamics.

In a study by Speck et al, trauma team participants described attributes of “good leaders” to be confidence, ability to remain calm, having the respect of team members, and clinical abilities.^2^ Medical students described good leaders as those with intelligence and experience and those who taught well.^2^ In our study, male respondents, physicians, and self-identified team leaders all gave higher CSACD.T scores than female respondents, non-physicians, and general team members. Male leaders and male nurses gave higher raw scores than female leaders and female nurses, respectively, but the differences were not statistically significant. The gender differences might be attributable to women feeling less heard during an intense situation such as a trauma resuscitation and attempting to avoid being perceived negatively if they become aggressive and violate expected norms of gender behavior.^16^

Multiple strategies have been employed to attain the goal of improving patient outcomes. Simulation and cross-disciplinary training fill gaps in care providers’ knowledge of traumatic injuries and diagnostic/stabilization procedures.^17^–^19^ Although increased attention has been directed toward communication, handoffs, and checklists in medicine, specific attention to training on how to function efficiently on ad hoc teams is lacking.

## LIMITATIONS

The study site is unique in that a dedicated trauma team evaluates and manages trauma patients; thus, team attitudes may vary at hospitals with different clinical practice. The survey instrument used in the study was reframed for the trauma team setting from the original validated CSACD instrument. The use of a self-administered survey has inherent limitations. Team members may have declined to participate due to being deeply unsatisfied, biasing the results. In future studies, a trained independent observer can be used to add objective measures of team dynamics.

Our study also lacked unique identifiers; so although attempts were made to avoid surveying the same individual twice during a shift, it is possible that multiple surveys were completed by one individual. Potential confounding factors that are independent of responder demographics yet can influence survey outcomes include severity of patient injury, patient outcome, time of day, postgraduate year of training and experience, and symptoms of burnout. This study did not attempt to link the CSACD.T scores of ad hoc trauma team members with patient outcomes. Larger, multicenter studies addressing similar questions may want to include patients’ characteristics and outcomes.

## CONCLUSION

Gender may appear to affect perceptions of collaboration and satisfaction with patient care decisions among trauma team members. This observation raises interesting questions about the underlying causes of those differences. Identification of the causes and their impact on trauma team collaboration and decision-making is an important next step.

## Supplementary Information



## Figures and Tables

**Table 1:** Comparison of self-identified demographic characteristics of individual respondents by gender (n = 281).

	Female (n = 111, 39.5%)	Male (n = 170, 60.5%)
Age		
20–30	49 (44.1)	70 (41.2)
31–40	40 (36)	70 (41.2)
41–45	4 (3.6)	11 (6.5)
46–55	12 (10.8)	8 (4.7)
56–65	6 (5.4)	1 (0.6)
>65	0 (0)	10 (5.9)
Ethnicity		
African American	3 (2.7)	15 (8.8)
Asian American	7 (6.3)	26 (15.3)
White	93 (83.8)	114 (67.1)
Hispanic	2 (1.8)	4 (2.4)
Native American	0 (0)	1 (0.6)
Other	6 (5.4)	10 (5.9)
Team leader	17 (15.3)	52 (30.6)
Occupation		
RN	48 (43.2)	30 (17.7)
Attending	6 (5.4)	15 (8.8)
Fellow	4 (3.6)	11 (6.5)
Resident	28 (25.2)	76 (44.7)
ED technician	17 (15.3)	24 (14.1)
Medical student	8 (7.2)	13 (7.7)
Other	0 (0)	1 (0.6)

*RN*, registered nurse; *ED*, emergency department.

**Table 2:** Demographics of trauma teams included in analysis of the Collaboration and Satisfaction About Care Decisions in Trauma survey (n = 52).

Characteristic	Number of teams (%)	Median (IQR)
No leader	14 (26.9)	
1 leader	21 (40.4)	
More than 1 leader	17 (32.7)	
Female leader	9 (17.3)	
Unknown leader gender	20 (38.5)	
All physician respondents	5 (9.6)	
All male respondents	7 (13.5)	
< 25% female respondents	11 (21.2)	
25%-50% female respondents	16 (30.8)	
50%–75% female respondents	18 (34.6)	
> 75% female respondents	7 (13.5)	
Size of team		6 (5, 8)
Response rate for team		62.5 (50.0, 75.0)
Percent of team male		58.6 (36.7, 75.0)
Percent of team white		75.0 (63.3, 100.0)
Percent of team physician		46.4 (33.3, 73.2)
Percent of team nurse		25.0 (7.1, 46.4)

*IQR*, interquartile range.

**Table 3:** Differences in individual responses to Collaboration and Satisfaction About Care Decisions in Trauma survey: mean scores* and 95% confidence intervals by gender, occupation, and team role.

Questions	Male N = 170	Female N = 111	*P-*value	Physician† N = 161	Nurse N = 78	*P-*value	Leader N = 61	Team Member N = 212	*p-*value
Q1: Nurses and physicians plan together to make decisions about care for the patients in the trauma bay	5.9 (5.8, 6.1)	5.7 (5.5, 5.9)	0.04	5.9 (5.8, 6.1)	5.7 (5.4, 6)	0.42	6.1 (5.9, 6.4)	5.8 (5.6, 5.9)	0.002
Q2: Open communication between physicians and nurses about patient care decisions takes place.	6.1 (6, 6.3)	5.9 (5.7, 6.1)	0.01	6.1 (6, 6.3)	5.8 (5.6, 6.1)	0.09	6.3 (6.1, 6.5)	6 (5.8, 6.1)	0.01
Q3: Decision-making responsibilities for patients are shared between nurses and physicians.	5.7 (5.5, 5.9)	5.5 (5.3, 5.7)	0.05	5.6 (5.4, 5.8)	5.5 (5.2, 5.8)	0.99	5.9 (5.6, 6.1)	5.6 (5.4, 5.7)	0.06
Q4: Physicians and nurses cooperate in making decisions about patient care.	6 (5.8, 6.1)	5.7 (5.5, 5.9)	0.01	5.9 (5.7, 6)	5.6 (5.4, 5.9)	0.27	6.1 (5.9, 6.3)	5.8 (5.6, 5.9)	0.02
Q5: In making decisions, both nursing and medical concerns about patients’ needs are considered.	6.1 (6, 6.3)	5.8 (5.6, 6)	0.002	6 (5.9, 6.2)	5.7 (5.5, 6)	0.09	6.2 (6.1, 6.4)	5.9 (5.7, 6)	0.02
Q6: Decision-making for patients is coordinated between physicians and nurses.	5.9 (5.7, 6)	5.5 (5.3, 5.7)	0.001	5.8 (5.6, 5.9)	5.5 (5.2, 5.8)	0.25	6.1 (5.8, 6.3)	5.6 (5.5, 5.8)	0.001
Q7: How much collaboration between nurses and physicians occurs when making patient care decisions?	5.8 (5.7, 6)	5.6 (5.4, 5.8)	0.02	5.8 (5.6, 5.9)	5.5 (5.2, 5.7)	0.15	6 (5.7, 6.2)	5.6 (5.5, 5.8)	0.02
Q8: How satisfied are you with the overall collaboration between physicians and nurses in the trauma bay?	6.1 (5.9, 6.2)	5.7 (5.6, 5.9)	0.003	6.1 (5.9, 6.2)	5.6 (5.3, 5.9)	0.01	6.2 (6, 6.3)	5.9 (5.7, 6)	0.05
Q9: How satisfied are you with collaboration on the trauma service overall?	6.1 (6, 6.2)	5.9 (5.7, 6.1)	0.03	6.1 (5.9, 6.2)	5.8 (5.5, 6)	0.11	6.2 (6, 6.3)	6 (5.8, 6.1)	0.26
Total score	54 (52.9, 55.1)	51.5 (50.1, 53)	0.001	53 (51.8, 54.2)	50.9 (48.9, 53)	0.14	55.2 (53.8, 56.6)	52.3 (51.2, 53.4)	0.01

* Scores based on a 7-point Likert scale.

† Physicians included attendings, fellows, and residents.
