# Supplementary material for: Collaboration and Decision-Making on Trauma Teams: A Survey Assessment
Source: West J Emerg Med. 2021 Jan 11;22(2):278–83. doi: 10.5811/westjem.2020.10.48698 (PMC7972389; doi:10.5811/westjem.2020.10.48698)
Supplement: Supplementary file 1 [file wjem-22-278-s001.docx]

**APPENDIX**

The Collaboration and Satisfaction About Care Decisions in Trauma survey consists of 9 questions, ranked on a 7-point Likert scale, relating to team collaboration as well as satisfaction with patient care decision-making.

**Collaboration and Satisfaction About Care Decisions in Trauma (CSACD.T)**

**Please respond to the following questions by circling your response. These questions are related to physician-nurse collaboration during patient care decision making. Please circle the number that best represents your judgment about the decision. All surveys are confidential.**

1. Nurses and physicians plan together to make decisions about care for the patients in the trauma bay.

1 2 3 4 5 6 7

Strongly disagree Strongly agree

2. Open communication between physicians and nurses about patient care decisions takes place.

1 2 3 4 5 6 7

Strongly disagree Strongly agree

3. Decision-making responsibilities for patients are shared between nurses and physicians.

1 2 3 4 5 6 7

Strongly disagree Strongly agree

4. Physicians and nurses cooperate in making decisions about patient care.

1 2 3 4 5 6 7

Strongly disagree Strongly agree

5. In making decisions, both nursing and medical concerns about patients’ needs are considered.

1 2 3 4 5 6 7

Strongly disagree Strongly agree

6. Decision-making for patients is coordinated between physicians and nurses.

1 2 3 4 5 6 7

Strongly disagree Strongly agree

7. How much collaboration between nurses and physicians occurs when making patient care decisions?

1 2 3 4 5 6 7

No Collaboration Complete Collaboration

8. How satisfied are you with the overall collaboration between physicians and nurses in the trauma bay?

1 2 3 4 5 6 7

Not satisfied at all Very satisfied

9. How satisfied are you with collaboration on the trauma service overall?

1 2 3 4 5 6 7

Not satisfied at all Very satisfied
